# Design, Synthesis and Anti-Tobacco Mosaic Virus (TMV) Activity of 5-Chloro-*N*-(4-cyano-1-aryl-1*H*-pyrazol-5-yl)-1-aryl-3-methyl-1*H*-pyrazole-4-carboxamide Derivatives

**DOI:** 10.3390/molecules20010807

**Published:** 2015-01-07

**Authors:** Jin-Jing Xiao, Min Liao, Ming-Jie Chu, Zi-Li Ren, Xin Zhang, Xian-Hai Lv, Hai-Qun Cao

**Affiliations:** 1 College of Plant Protection, Anhui Agricultural University, Hefei 230036, China; E-Mails: xiaojj187012@163.com (J.-J.X.); liaomin3119@126.com (M.L.); chumingjie@ahau.edu.cn (M.-J.C.); renzilix@163.com (Z.-L.R.); 2 State Key Laboratory of Pharmaceutical Biotechnology, Nanjing University, Nanjing 210093, China; E-Mail: zhangxinzxn@126.com

**Keywords:** pyrazole amide, synthesis, anti-TMV, molecule docking

## Abstract

A series of novel pyrazole amide derivatives **3a**–**3p** which take TMV PC protein as the target has been designed and synthesized by the reactions of 5-chloro-1-aryl-3-methyl-1*H*-pyrazole-4-carboxylic acids with 5-amino-1-aryl-1*H*-pyrazole-4-carbonitriles. All the compounds were characterized by ^1^H-NMR, mass spectroscopy and elemental analysis. Preliminary bioassays indicated that all the compounds acted against the tobacco mosaic virus (TMV) with different *in vivo* and *in vitro* modes at 500 μg/mL and were found to possess promising activity. Especially, compound **3p** showed the most potent biological activity against tobacco mosaic virus (TMV) compared to ningnanmycin, and a molecular docking study was performed and the binding model revealed that the pyrazole amide moiety was tightly embedded in the binding sites of TMV PC (PDB code: 2OM3).

## 1. Introduction

Tobacco mosaic virus (TMV) is one of the most extensively studied among a number of plant viruses. Its infections are very widely distributed [1,2,3] and can cause serious damage and large economic losses. TMV is a growing problem in the contemporary pesticide field, as only a few antiviral agents are currently available in practice, and almost no treatment can control TMV effectively [4], so the identification of new antiviral agent with novel mechanisms of action is critically needed to overcome this viral infections.

Recently, different targets essential to plant viruses that could be new weapons to design and discover new antiviral drugs have been studied and validated. Most antiviral drugs exhibit their activities through interaction with viral proteins [5,6]. Coat protein-mediated resistance to TMV involves mainly two independent mechanisms: (i) interference by transgenic CP with disassembly of challenge virus; and (ii) interference of transgenic CP with formation of replication complexes, thus interfering with virus movement [7]. CP is an important part of the genomic structure of the tobacco mosaic virus (TMV), and its main function not only includes the protection of DNA from degradation, but it also has a close relationship with the long-distance movement of TMV and host symptoms [8]. Therefore, coat protein (PC) is usually used as a molecular target for drug action, and the study of TMV CP mainly focuses on CP-mediated resistance, interference the polymerization of CP by agents and so on. Many small molecule ligands. including pyrazole derivatives, that combine with molecular targets have been reported [9,10,11,12]. Inspired by the above notion, we hope to design a new compound which is able to target TMV PC to inhibit viral assembly through small molecule-coat protein interactions.

**Figure 1:**
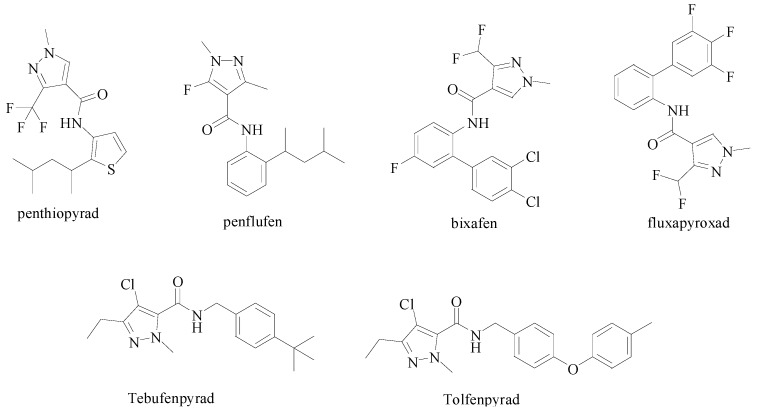
Pyrazole derivative products.

Pyrazole derivatives have great importance in the medicinal and pesticide fields [13,14,15], due to their broad range of biological activity [16,17,18]. The molecules of many modern drugs with antiphlogistic, antidiabetic, analgesic, and acaricidal activity in medicine [19] and herbicidal [20,21,22], antibacterial [23,24,25], acaricidal and insecticide [26,27] in the pesticide area contain pyrazole rings as a structural fragment. As shown in Figure 1, several pyrazole derivative products have been launched or announced, and they all contain several common structural features that are essential to their activity, including an amide bond, pyrazole and an aromatic ring attached to the carbonyl of the amide bond. 

**Figure 2:**
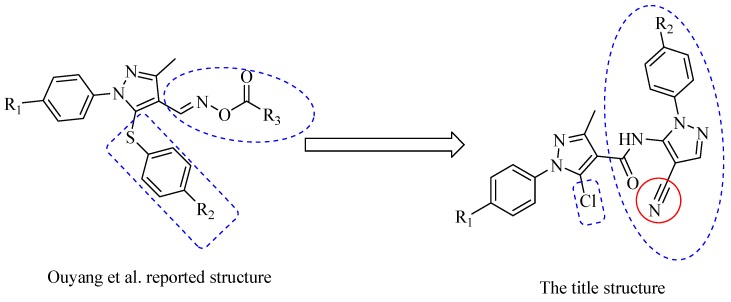
Design of the target compounds.

Modification of the structural profile by the change of substituents at the 1-, 3-, or 4-positions in the pyrazole ring can bring about significant changes in bioactivity [28], and linking the pyrazole group with a structurally diverse side chain is an effective way to obtain new heterocyclic derivatives with high antiviral activities [29]. We assumed that if nitrile-containing pyrazole pharmacophores were introduced into the pyrazole scaffold, thus transforming the structure reported by Ouyang *et al.* [30], the resulting compounds may be interesting lead structures for antiviral agent development (Figure 2), because the nitrile can modulate the physicochemical and pharmacokinetic properties to improve bioavailability, enhance the selectivity and binding affinity to target proteins by hydrogen bond interactions, covalent interactions, polar interactions, and π-π interactions [31,32,33], and cyano substitution in the design of drugs has become one of the important research strategies in optimizing the structure of lead compounds [34]. 

**Figure 3:**
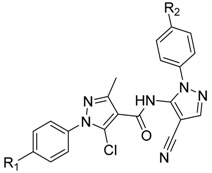
The structure of compounds **3a**–**3p**.

**Figure 4:**
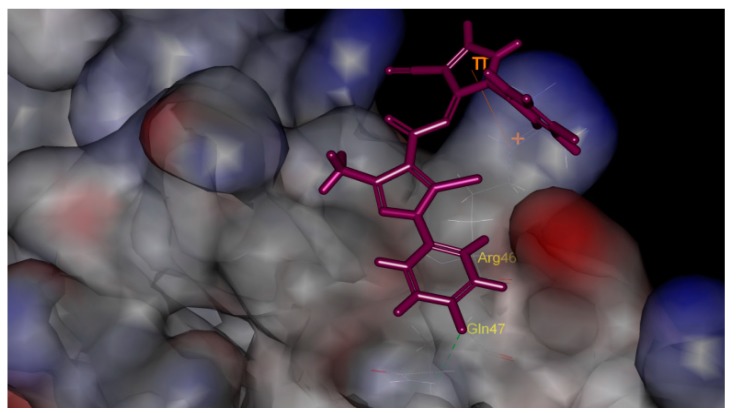
Docking model of compound **3p** with TMV PC. It is nicely bound to the TMV PC one H-bond (GIn47-F:2.49 Å, 109.02°) and has a π-cation interaction (4.65 Å).

We describe herein the synthesis and antiviral activity of a series of new pyrazole amide derivatives (Figure 3). Molecular docking was performed and the binding model revealed that the title pyrazole amide was tightly embeded in the binding sites of TMV PC (PDB code: 2OM3) (Figure 4). The anti-TMV activities of these compounds were subsequently evaluated, and a structure-activity relationship was established in terms of their anti-TMV activity.

**Table:** 

Compound NO.	R_1_	R_2_
**3a**	H	H
**3b**	H	Cl
**3c**	H	CH_3_
**3d**	H	F
**3e**	Cl	H
**3f**	Cl	Cl
**3g**	Cl	CH_3_
**3h**	Cl	F
**3i**	CH_3_	H
**3j**	CH_3_	Cl
**3k**	CH_3_	CH_3_
**3l**	CH_3_	F
**3m**	F	H
**3n**	F	Cl
**3o**	F	CH_3_
**3p**	F	F

## 2. Results and Discussion

### 2.1. Chemistry

**Scheme 1:**
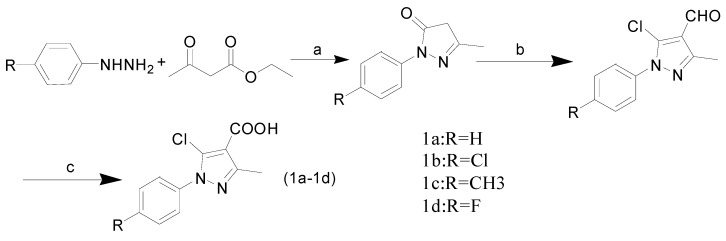
General synthesis of compounds **1a**–**1d**. (**a**) H_2_O, ethanol, 60 °C, TLC; (**b**) DMF, POCl_3_, 80–85 °C, TLC; (**c**) KMnO_4_, 70–80 °C, TLC.

**Scheme 2:**
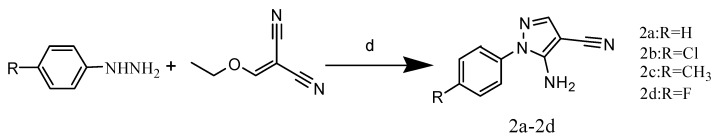
General synthesis of compounds **2a**–**2d**. (**d**) H_2_O, NaOH, ethanol, 3 h, reflux.

**Scheme 3:**
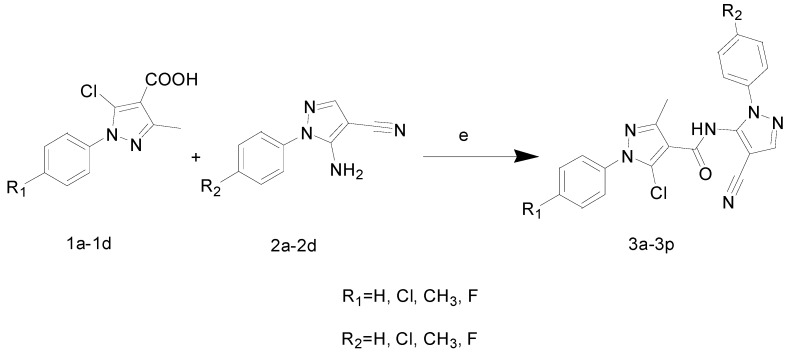
General synthesis of compounds **3a**–**3p**. (**e**) DMF, EDCI, HOBt, rt, TLC.

The synthetic route is shown in Scheme 1, Scheme 2 and Scheme 3. Compounds **1a**–**1d** were prepared in accordance with the literature procedure [35] from the reactions of substituted phenylhydrazines with ethyl acetoacetate in anhydrous ethanol medium. Then the reaction mixtures was added to a cold solution of DMF and POCl_3_. Finally, the products were oxidized with KMnO_4_, to give the 5-chloro-1-aryl-3-methyl-1*H*-pyrazole-4-carboxylic acids **1** (Scheme 1).

Compounds **2a**–**2d** were obtained from substituted phenyl hydrazine hydrochlorides and 2-(ethoxymethylene) malononitrile in ethanol medium, which was refluxed for 3 h to produce the 5-amino-1-aryl-1*H*-pyrazole-4-carbonitriles **2a**–**2d** [36] (Scheme 2).

The compounds **1** and **2** were reacted using 1-ethyl-3-(3-dimethylaminopropyl) carbodiimide hydrochloride (EDCI) and N-hydroxybenzotriazole (HOBt) in DMF medium in the presence of triethylamine as catalyst at room temperature to produce the 5-chloro-N-(4-cyano-1-aryl-1*H*-pyrazol-5-yl)-1-aryl-3-methyl-1*H*-pyrazole-4-carboxamides **3a**–**3p** (Scheme 3).

The structures of the newly synthesized compounds were confirmed by ^1^H-NMR, mass spectroscopy and elemental analysis. In the ^1^H-NMR (CDCl_3_) spectra of the compounds, the N-H proton was hard to distinguish in the spectra. To prove its existence we used DMSO as solvent to dissolve compounds **3e**, **3f**, **3n** and then recorded the NMR spectra, whereupon the signal of compounds **3e**, **3n** appears at 12.88 ppm and the signal of compounds **3f** appears at 12.89 ppm. The NH group of compound **3n** appears at 115.11 ppm in the corresponding ^13^C-NMR spectrum taken in DMSO. The IR spectrum of compound **3b** has been investigated in detail to exactly indicate the presence of CN groups and the main observed absorption bands were those located at: 3180.66 cm^−1^ (N-H), 2230.44 cm^−1^ (C≡N), 1636.96 cm^−1^ (C=O), 1560.03 cm^−1^ (C=O), 1404.77 cm^−1^ (C-N), 821.27 cm^−1^ (C-Cl arom), and 587.59 cm^−1^ (C-Cl pyrazole), which all support the proposed structure. 

### 2.2. Bioactivity

To judgme the antiviral potency of the synthesized compounds **3a**–**3p**, the commercially available plant virucide ningnanmycin was used as the control. The antiviral bioassay against TMV was assayed by the method reported by Thorson *et al.* [37]. 

The *in vivo* and *in vitro* bioassay results of compounds **3a**–**3p** against TMV are given in Table 1. The results indicated that the title compounds showed curative rates ranging from 22.6%–86.5%. Among them, compound **3p** showed the most potent biological activity against TMV and it exhibited slightly higher activities compared to the commercial agent ningnanmycin.

**Table 1:** Anti-TMV activity of compounds *in vitro* and *in vivo* at 500 mg/mL.

Compound NO.	*In Vitro* Inhibition Rate (%)	*In Vivo* Inhibition Rate (%)		
		Protection Effect	Inactivation Effect	Curative Effect
**3a**	54.8 ± 1.11	32.4 ± 1.45	43.2 ± 4.01	47.3 ± 0.88
**3b**	66.4 ± 0.78	48.4 ± 0.23	54.2 ± 2.11	54.5 ± 1.03
**3c**	52.1 ± 1.20	43.1 ± 2.33	42.6 ± 1.09	42.2 ± 2.12
**3d**	68.1 ± 2.33	60.2 ± 2.11	53.1 ± 2.09	55.1 ± 2.15
**3e**	67.2 ± 0.48	49.3 ± 1.03	58.4 ± 0.97	56.5 ± 0.55
**3f**	83.2 ± 1.16	76.7 ± 2.54	74.2 ± 1.58	78.5 ± 0.82
**3g**	64.3 ± 0.66	33.8 ± 3.56	52.3 ± 3.12	41.2 ± 3.20
**3h**	81.6 ± 0.72	78.9 ± 1.21	66.4 ± 4.23	75.3 ± 1.31
**3i**	53.3 ± 0.51	22.6 ± 1.36	43.3 ± 1.24	52.1 ± 2.11
**3j**	42.3 ± 0.48	45.2 ± 1.97	52.6 ± 0.65	44.2 ± 5.12
**3k**	50.2 ± 1.33	42.1 ± 1.29	22.8 ± 6.03	38.9 ± 4.33
**3l**	64.6 ± 4.11	41.2 ± 7.03	45.3 ± 7.34	42.1 ± 1.43
**3m**	74.4 ± 2.08	52.1 ± 3.02	64.6 ± 0.63	45.9 ± 3.32
**3n**	84.3 ± 1.52	77.5 ± 3.51	74.5 ± 2.13	78.9 ± 1.65
**3o**	72.1 ± 1.18	61.3 ± 1.30	67.8 ± 0.92	52.1 ± 2.06
**3p**	86.5 ± 4.96	77.2 ± 6.03	84.3 ± 0.52	81.5 ± 1.03
**Ningnanmycin**	82.1 ± 3.31	62.4 ± 7.20	78.4 ± 5.14	55.2 ± 6.12

### 2.3. Molecular Docking

Molecular docking of compound **4p** into the three dimensional X-ray structure of the TMV PC protein-ligand complex crystal structure (PDB code: 2OM3) was carried out using the Discovery Studio (DS) (version 3.1, NeoTrident Corporation, Beijing, China) software [38] as implemented through the graphical user interface DS-CDOCKER protocol. The three-dimensional structures of the aforementioned compounds were constructed using Chem. 3D ultra 12.0 software (Chemical Structure Drawing Standard, CambridgeSoft Corporation, Cambridge, MA, USA), then they were energetically minimized by using MMFF94 with 5000 iterations and minimum RMS gradient of 0.10. The crystal structures of protein complex were retrieved from the RCSB Protein Data Bank (http://www.rcsb.org/pdb/home/home.do) and prepared by Discovery Studio 3.1 with all bound waters and ligands eliminated from the protein and the polar hydrogen added to the protein. The molecular docking procedure was performed by using CDOCKER protocol for receptor-ligand interactions section of DS 3.1.

### 2.4. 3D-QSAR

**Figure 5:**
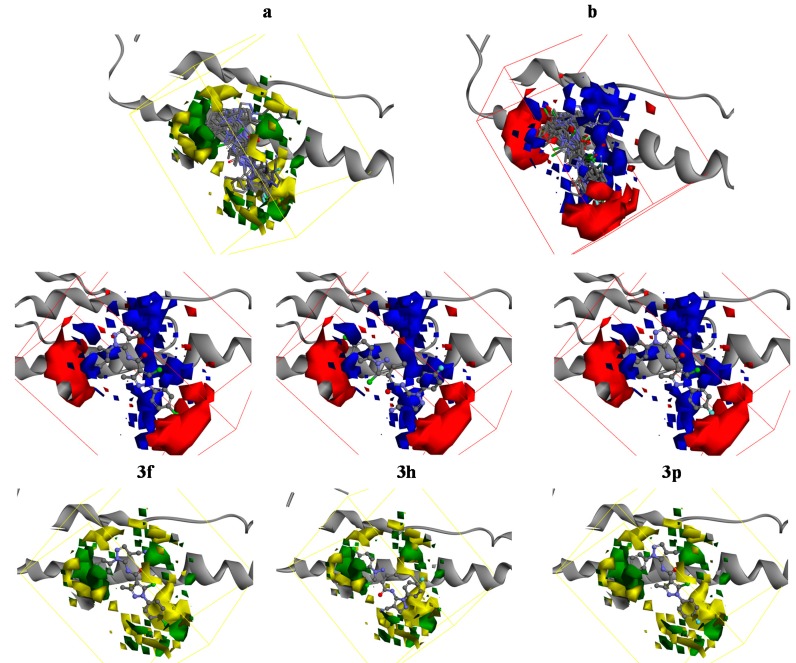
3D-QSAR of pyrazole amide derivatives for TMV PC (PDB: 2OM3). (**a**) 3D-QSAR model coefficients on van der Waals grids. Green represents positive coefficients; yellow represents negative coefficients; (**b**) 3D-QSAR model coefficients on electrostatic potential grids. Blue represents positive coefficients; red represents negative coefficients.

In order to further explain of structure and activity relationships of these compounds, the *in vitro* antiviral activity data were used to build the 3D-QSAR models, and the 3D-QSAR study was performed by means of the DS 3.5 software. The correlation coefficient r^2^ between observed and predicted activity of training set was found to be 0.899, which meant the dependability of linear fit. The molecules aligned with the iso-surfaces of the 3D-QSAR model coefficients on electrostatic potential grids (Figure 5a) and van der Waals grids (Figure 5b) are listed. 

As shown in Figure 5, compounds with electron-withdrawing atoms not only circumvented the red subregion but also got more close to the favorable green spaces. This figure showed that a halogen substituted group was a better choice than a methyl substituted group and F was a better substituent than Cl, so this QSAR study could predict that electron-withdrawing groupa substituted in the aromatic ring could have good interaction with the protein with nice molecule electrostatic and steric features. However, the chlorine atom is also a strong electron withdrawing-group but its ability is slight weaker than that of a F atom, therefore, the compounds with Cl atom substituted in the aromatic ring (e.g., **3f**, **3h**) have similar inhibition as **3p**. The 3D QSAR models fitted the inhibitory activity well and thus provide us the directions for further modification.

## 3. Experimental Section

### 3.1. General Information

Melting points (uncorrected) were determined on an XT4 MP apparatus (Taike Corp., Beijing, China). ESI mass spectra were obtained on a Mariner System 5304 mass spectrometer (Applied Biosystems, Foster City, CA, USA). ^1^H-NMR spectra were recorded on a Bruker PX400 spectrometer (Bruker Corporation, Rheinstetten, Germany) at 25 °C with TMS and solvent signals assigned as internal references, ^13^C-NMR spectra and ^1^H-NMR spectra of compound **3e**, **3n** were recorded on an Agilent DD2 PX600 spectrometer (Agilent Technologies Corporation, Santa Clara, CA, USA). Chemical shifts were reported in ppm (δ). Elemental analyses were performed on a CHN-O-Rapid instrument (Leco, Tres Cantos, Madrid, Spain) and were within 0.4% of the theoretical values.

### 3.2. General Procedure for Synthesis of 5-Chloro-1-aryl-3-methyl-1H-pyrazole-4-carboxylic Acids **1a**–**1d**

The intermediate 5-chloro-1-aryl-3-methyl-1*H*-pyrazole-4-carboxylic acids **1a**–**1d** were synthesized as the following: *para*-substituted phenylhydrazines (0.025 mol) were reacted with ethyl acetoacetate (0.025 mol) in anhydrous ethanol to form a solid, which was then dissolved in a cold mixed solution of DMF (20 mL) and POCl_3_ (16 mL), and stirred at 50–60 °C. The resulting mixture was poured into ice-cold water, a saturated solution of sodium hydroxide was added to neutralize the mixture, and the solid precipitate was filtered, and washed with water. Then above product was oxidized by KMnO_4_ solution, stirred at 70–80 °C. After cooling to room temperature the pH of the reaction mixture was adjusted to pH 7–8 by the dropwise addition of KOH solution, and the solution was filtered, HCl solution was added to the solution and solid **1a**–**1d** eventually separated out. The crude product obtained was recrystallized from DMF to afford the pure product. All the reactions were monitored by TLC.

### 3.3. General Procedure for Synthesis of 5-Amino-1-aryl-1H-pyrazole-4-carbonitriles **2a**–**2d**

A stirred mixture of *para*-substituted phenylhydrazine hydrochloride (0.025 mol) was dissolved in H_2_O (30 mL), then the pH of the mixture was adjusted to pH 7–8 by the dropwise addition of 10% NaOH solution to form the free *para*-substituted phenyl hydrazines, which were then refluxed for 3 h with ethoxymethylene malononitrile in an ethanol medium. After completion of the reaction, the reaction mixture was allowed to cool at room temperature, and the solid **2a**–**2d** were filtered under vacuum. The crude products obtained were recrystallized from DMF to afford the pure products. 

### 3.4. General Procedure for Synthesis of 5-Chloro-N-(4-cyano-1-aryl-1H-pyrazol-5-yl)-1-aryl-3-methyl-1H-pyrazole-4-carboxamide Derivatives **3a**–**3p**

To a stirred solution of the intermediate compounds **1a**–**1d** (1 mmol) and triethylamine (2 mmol) in DMF (12 mL) medium, a mixture of EDCI (1 mmol) and HOBt (1mmol) was added and the reaction mixture was stirred at room temperature for 30 min, then a mixture of compounds **2a**–**2d** (1 mmol) and DMF (5 mL) was added, the reaction was stirred at room temperature. And the reaction progress was monitored by TLC. After completion of the reaction, the product was added into chloroform, then extracted from chloroform with water, and washed successively with 0.2 mol/L hydrochloric acid, water, 2 mol/L sodium hydroxide, water, saturated sodium chloride, then dried, concentrated and purified by preparative thin layer chromatography (PE:EA = 8:1) followed by recrystallization from ethanol.

*5-Chloro-N-(4-cyano-1-phenyl-1H-pyrazol-5-yl)-3-methyl-1-phenyl-1H-pyrazole-4-carboxamide* (**3a**). White crystals, yield 68%, mp: 133–135 °C. ^1^H-NMR (400 MHz, CDCl_3_) δ: 8.12 (s, 1H), 7.61–7.43 (m, 10H), 2.66 (s, 3H). MS (ESI): 403.1 (C_21_H_15_ClN_6_O, [M+H]^+^). Anal. Calcd for C_21_H_15_ClN_6_O: C, 62.61; H, 3.75; N, 20.86; Found: C, 62.82; H, 3.84; N, 21.04.

*5-Chloro-1-(4-chlorophenyl)-N-(4-cyano-1-phenyl-1H-pyrazol-5-yl)-3-methyl-1H-pyrazole-4-carbox-amide* (**3b**). White crystals, yield 68%. mp: 171–172 °C, IR (cm^−1^): 3180.66, 2230.44, 1636.96, 1560.03, 1404.77, 821.27, 587.59. ^1^H-NMR (400 MHz, CDCl_3_) δ: 8.12 (s, 1H), 7.55–7.43 (m, 9H), 2.65 (s, 3H). MS (ESI): 437.1 (C_21_H_14_Cl_2_N_6_O, [M+H]^+^). Anal. Calcd for C_21_H_14_Cl_2_N_6_O: C, 57.68; H, 3.23; N, 19.22; Found: C, 57.72; H, 3.31; N, 19.11.

*5-Chloro-N-(4-cyano-1-(p-tolyl)-1H-pyrazol-5-yl)-3-methyl-1-phenyl-1H-pyrazole-4-carboxamide* (**3c**). White crystals, yield 62%, mp: 123–125 °C; ^1^H-NMR (400 MHz, CDCl_3_) δ: 8.12 (s, 1H), 7.63–7.41 (m, 9H), 2.66 (s, 3H), 2.42 (s, 3H). MS (ESI): 417.1 (C_22_H_17_ClN_6_O, [M+H]^+^). Anal. Calcd for C_22_H_17_ClN_6_O: C, 63.39; H, 4.11; N, 20.16; Found: C, 63.71; H, 4.42; N, 20.22.

*5-Chloro-N-(4-cyano-1-(4-fluorophenyl)-1H-pyrazol-5-yl)-3-methyl-1-phenyl-1H-pyrazole-4-carbox-amide* (**3d**). White crystal, yield 71%, mp: 181–183 °C. ^1^H-NMR (400 MHz, CDCl_3_) δ: 8.12 (s, 1H), 7.51–7.21 (m, 9H), 2.66 (s, 3H). MS (ESI):421.0 (C_21_H_14_ClFN_6_O, [M+H]^+^). Anal. Calcd for C_21_H_14_ClFN_6_O: C, 59.54; H, 3.35; N, 19.97; Found: C, 59.72; H, 3.41; N, 20.06.

*5-Chloro-1-(4-chlorophenyl)-N-(4-cyano-1-phenyl-1H-pyrazol-5-yl)-3-methyl-1H-pyrazole-4-carbox-amide* (**3e**). White crystals, yield 66%, mp: 172–174 °C. ^1^H-NMR (400 MHz, CDCl_3_) δ: 8.12 (s, 1H), 7.61–7.43 (m, 9H), 2.65 (s, 3H). ^1^H-NMR (600 MHz, DMSO-*d_6_*) δ: 12.88 (s, 1H), 7.97 (s, 1H), 7.62–7.41 (m, 9H), 2.48 (s, 3H). MS (ESI): 437.1 (C_21_H_14_Cl_2_N_6_O, [M+H]^+^). Anal. Calcd for C_21_H_14_Cl_2_N_6_O: C, 57.68; H, 3.23; N, 19.22; Found: C, 57.74; H, 3.41; N, 19.42.

*5-Chloro-1-(4-chlorophenyl)-N-(1-(4-chlorophenyl)-4-cyano-1H-pyrazol-5-yl)-3-methyl-1H-pyrazole-4-carboxamide* (**3f**). White crystals, yield 79%, mp: 178–180 °C. ^1^H-NMR (400 MHz, DMSO-*d_6_*) δ: 12.89 (s, 1H), 7.98 (s, 1H), 7.63–7.42 (m, 8H), 2.65 (s, 3H). MS (ESI): 471.0 (C_21_H_13_Cl_3_N_6_O, [M+H]^+^). Anal. Calcd for C_21_H_13_Cl_3_N_6_O: C, 53.47; H, 2.78; N, 17.82; Found: C, 53.56; H, 2.81; N, 17.91.

*5-Chloro-1-(4-chlorophenyl)-N-(4-cyano-1-(p-tolyl)-1H-pyrazol-5-yl)-3-methyl-1H-pyrazole-4-carboxamide* (**3g**). White crystals, yield 69%, mp: 130–132 °C. ^1^H-NMR (400 MHz, CDCl_3_) δ: 8.12 (s, 1H), 7.60–7.43 (m, 8H), 2.65 (s, 3H), 2.42 (s, 3H). MS (ESI): 451.1 (C_22_H_16_Cl_2_N_6_O, [M+H]^+^). Anal. Calcd for C_22_H_16_Cl_2_N_6_O: C, 58.55; H, 3.57; N, 18.62; Found: C, 58.58; H, 3.71; N, 18.74.

*5-Chloro-1-(4-chlorophenyl)-N-(4-cyano-1-(4-fluorophenyl)-1H-pyrazol-5-yl)-3-methyl-1H-pyrazole-4-carboxamide* (**3h**). White crystals, yield 69%, mp: 177–178 °C. ^1^H-NMR (400 MHz, CDCl_3_) δ: 8.12 (s, 1H), 7.53–7.20 (m, 8H), 2.66 (s, 3H). MS (ESI): 455.1 (C_21_H_13_Cl_2_FN_6_O, [M+H]^+^). Anal. Calcd for C_21_H_13_Cl_2_FN_6_O: C, 55.40; H, 2.88; N, 18.46; Found: C, 55.51; H, 2.93; N, 18.48.

*5-Chloro-N-(4-cyano-1-phenyl-1H-pyrazol-5-yl)-3-methyl-1-(p-tolyl)-1H-pyrazole-4-carboxamide* (**3i**). White crystals, yield 64%, mp: 124–126 °C. ^1^H-NMR (400 MHz, CDCl_3_) δ: 8.12 (s, 1H), 7.62–7.41 (m, 9H), 2.65 (s, 3H), 2.42 (s, 3H). MS (ESI): 417.1 (C_22_H_17_ClN_6_O, [M+H]^+^). Anal. Calcd for C_22_H_17_ClN_6_O: C, 63.39; H, 4.11; N, 20.16; Found: C, 63.44; H, 4.14; N, 20.18.

*5-Chloro-N-(1-(4-chlorophenyl)-4-cyano-1H-pyrazol-5-yl)-3-methyl-1-(p-tolyl)-1H-pyrazole-4-carboxamide* (**3j**). White crystals, yield 77%, mp: 155–157 °C. ^1^H-NMR (400 MHz, CDCl_3_) δ: 8.11 (s, 1H), 7.61–7.41 (m, 8H), 2.65 (s, 3H), 2.46 (s, 3H). MS (ESI): 451.1 (C_22_H_16_Cl_2_N_6_O, [M+H]^+^). Anal. Calcd for C_22_H_16_Cl_2_N_6_O: C, 58.55; H, 3.57; N, 18.62; Found: C, 58.58; H, 3.61; N, 18.66.

*5-Chloro-N-(4-cyano-1-(p-tolyl)-1H-pyrazol-5-yl)-3-methyl-1-(p-tolyl)-1H-pyrazole-4-carboxamide* (**3k**). White crystals, yield 63%, mp: 133–135 °C. ^1^H-NMR (400 MHz, CDCl_3_) δ: 8.12 (s, 1H), 7.61–7.43 (m, 8H), 2.65 (s, 3H), 2.42 (s, 6H). MS (ESI): 431.1 (C_23_H_19_ClN_6_O, [M+H]^+^). Anal. Calcd for C_23_H_19_ClN_6_O: C, 64.11; H, 4.44; N, 19.50; Found: C, 64.17; H, 4.47; N, 19.56.

*5-Chloro-N-(4-cyano-1-(4-fluorophenyl)-1H-pyrazol-5-yl)-3-methyl-1-(p-tolyl)-1H-pyrazole-4-carbox-amide* (**3l**). White crystals, yield 62%, mp: 156–158 °C. ^1^H-NMR (400 MHz, CDCl_3_) δ: 8.12 (s, 1H), 7.61–7.22 (m, 8H), 2.65 (s, 3H), 2.42 (s, 3H). MS (ESI): 435.1 (C_22_H_16_ClFN_6_O, [M+H]^+^). Anal. Calcd for C_22_H_16_ClFN_6_O: C, 60.76; H, 3.71; N, 19.33; Found: C, 60.77; H, 3.74; N, 19.46.

*5-Chloro-N-(4-cyano-1-phenyl-1H-pyrazol-5-yl)-1-(4-fluorophenyl)-3-methyl-1H-pyrazole-4-carbox-amide* (**3m**). White crystals, yield 69%, mp: 118–120 °C. ^1^H-NMR (400 MHz, CDCl_3_) δ: 8.12 (s, 1H), 7.61–7.23 (m, 9H), 2.65 (s, 3H). MS (ESI): 421.1 (C_21_H_14_ClFN_6_O, [M+H]^+^). Anal. Calcd for C_22_H_16_ClN_6_O: C, 59.94; H, 3.35; N, 19.97; Found: C, 60.07; H, 3.43; N, 20.04.

*5-Chloro-N-(1-(4-chlorophenyl)-4-cyano-1H-pyrazol-5-yl)-1-(4-fluorophenyl)-3-methyl-1H-pyrazole-4-carboxamide* (**3n**). White crystals, yield 81%, mp: 149–151 °C. ^1^H-NMR (400 MHz, CDCl_3_) δ: 8.12 (s, 1H), 7.62–7.22 (m, 8H), 2.65 (s, 3H). ^1^H-NMR (600 MHz, DMSO-*d_6_*) δ: 12.88 (s, 1H), 7.97 (s, 1H), 7.62–7.21 (m, 8H), 2.48 (s, 3H). ^13^C-NMR (151 MHz, DMSO-*d*_6_) δ 163.47, 163.14, 151.88, 151.73, 142.43, 136.76, 133.97, 132.68, 131.25, 128.64, 128.58, 116.71, 116.56, 115.11, 110.46, 73.96, 14.86. MS (ESI): 455.1 (C_21_H_13_Cl_2_FN_6_O, [M+H]^+^). Anal. Calcd for C_21_H_13_Cl_2_FN_6_O: C, 55.40; H, 2.88; N, 18.46; Found: C, 55.53; H, 2.91; N, 18.49.

*5-Chloro-N-(4-cyano-1-(p-tolyl)-1H-pyrazol-5-yl)-1-(4-fluorophenyl)-3-methyl-1H-pyrazole-4-carbox-amide* (**3o**). White crystals, yield 65%, mp: 127–129 °C. ^1^H-NMR (400 MHz, CDCl_3_) δ: 8.12 (s, 1H), 7.61–7.22 (m, 8H), 2.65 (s, 3H), 2.42 (s, 3H). MS (ESI): 435.1 (C_22_H_16_ClFN_6_O, [M+H]^+^). Anal. Calcd for C_22_H_16_ClFN_6_O: C, 60.76; H, 3.71; N, 19.33; Found: C, 60.80; H, 3.77; N, 19.49.

*5-Chloro-N-(4-cyano-1-(4-fluorophenyl)-1H-pyrazol-5-yl)-1-(4-fluorophenyl)-3-methyl-1H-pyrazole-4-carboxamide* (**3p**). White crystals, yield 62%, mp: 182–183 °C. ^1^H-NMR (400 MHz, CDCl_3_) δ: 8.12 (s, 1H), 7.61–7.21 (m, 8H), 2.65 (s, 3H). MS (ESI): 439.1 (C_21_H_13_ClF_2_N_6_O, [M+H]^+^). Anal. Calcd for C_21_H_13_ClF_2_N_6_O: C, 57.48; H, 2.99; N, 19.15; Found: C, 57.53; H, 3.03; N, 19.17.

### 3.5. Antiviral Biological Assay

The anti-TMV activity of the synthesized pure compounds were tested by using the method reported by Thorson *et al.* [37]. This method is to measure the antiviral activity of compounds *in vitro* and to test the protective effect, the inactivation effect and the curative effect *in vivo* against TMV.

#### 3.5.1. Antiviral Activity of Target Compounds *in Vitro*

Fresh 5–6 growth stage of tobacco leaves (*Nicotianatabacum* var. *Xanthi*NC) were selected for the test. The tobacco was inoculated by the juice-leaf rubbing method, and the concentration of TMV was 5.88 × 10^−2^ μg/mL. The leaves was cut into halves along the main vein, and a solution of the compounds with a concentration of 500 μg/mL was smeared on the halves, and then cultured at 25 °C for 72 h. Each compound was tested three times.

#### 3.5.2. Protective Effect of Target Compounds against TMV *in Vivo*

The test compound solution was smeared on the left side, and the solvent served as control on the right side of growing tobacco (*Nicotianatabacum* var. *XanthiNC*). After 12 h, TMV at a concentration of 6.0 μg/mL was inoculated on the leaves which were previously scattered with silicon carbide through the above juice-leaf rubbing method. Then the leaves were again immersed into water and rubbed softly along the nervation once or twice. The local lesion numbers showing 3–4 days after inoculation were counted. Each compound was tested in three replicates.

#### 3.5.3. Inactivate Effect of Target Compounds against TMV *in Vivo*

The virus was inhibited by mixing with the target compound solution at the same volume for 30 min. Then the mixture was inoculated on the left side of the host tobacco leaves (*Nicotianatabacum* var. *XanthiNC*), and a mixture of solvent and the virus was inoculated on the right side to serve as control. The local lesion numbers showing 3–4 days after inoculation were counted. Each compound was tested in three replicates.

#### 3.5.4. Curative Effect of Target Compounds against TMV *in Vivo*

Host plant tobacco leaves (*Nicotianatabacum* var. *XanthiNC*) of the same age growing at the six-leaf stage were selected for the test. TMV at a concentration of 6.0 μg/mL was inoculated on the whole leaves. The leaves were washed with water, and dried in a greenhouse. The target compound solution was smeared on the left side, and the solvent served as control on the right side. The local lesion numbers showing 3–4 days after inoculation were counted. Each compound was tested in three replicates. The inhibition rates of the compound *in vitro* and *in vivo* were calculated according to the following formula (controls were not treated with compound):

Inhibition rate (%) = [(average local lesion number of control − average local lesion number of drug treated)/average local lesion number of control] × 100%
(1)

## 4. Conclusions

In conclusion, a series of novel pyrazole amide compounds has been designed and synthesized, based on the skeleton of previously reported pyrazole derivatives. The title compounds **3a**–**3p** were evaluated as potent antiviral agents against TMV and it was proved that compound **3p** showed a significant activity.

Structure-activity relationships in these pyrazole amide derivatives were evaluated using the DS approach to proceed with structure optimization, The data demonstrated that compounds with *para* electron-withdrawing substituents (e.g., **3f**, **3h**, **3n**, **3p**) in the A-ring showed more potent activities than those with electron-donating substituents (e.g., **3a**, **3d**, **3i**, **3k**). In comparison the activity of compound with *para* substituents on the phenyl ring showed that an electron-withdrawing group gave slightly improved antiviral activity and the potency order is F > Cl. Among all the compounds, **3p** with a *para*-F group showed more activity, while **3k** showed the lowest activity.

For the binding model of compound **3p**, an H-bond (2.49 Å and 109.02°) was established between its fluorine atom and Gin47, and it also formed a π-cation interaction (4.65 Å). The result of molecular docking suggests that introduction of a fluorine atom facilitates the adoption of a complementary shape of the designed ligands with TMV PC, and this possibly improves the anti-TMV inhibitory potency.
